# Supplementary material for: Modular assembly of self-healing flexible thermoelectric devices with integrated cooling and heating capabilities
Source: Nat Commun. 2025 May 7;16:4220. doi: 10.1038/s41467-025-59602-8 (PMC12056181; doi:10.1038/s41467-025-59602-8)
Supplement: Supplementary file 1 — Supplementary Information [file 41467_2025_59602_MOESM1_ESM.pdf]

Supplementary Materials for

Modular Assembly of Self-healing Flexible Thermoelectric Devices with Integrated Cooling and Heating Capabilities

Xiaolong Sun[1,2,3], Yue Hou[1,*], Zheng Zhu[1], Bo Zhu[1], Qianfeng Ding[1], Wenjie Zhou[1], Sijia Yan[1], Zhanglong Xia[1], Yong Liu[2], Youmin Hou[4,5] *, Yuan Yu[6,*], Ziyu Wang[1,2,3] *

[1]The Institute of Technological Sciences, Wuhan University, Wuhan 430072, China.

[2]Key Laboratory of Artificial Micro-structures of Ministry of Education, School of Physics and Technology, Wuhan University, Wuhan 430072, China.

[3]School of Physics and Microelectronics, Key Laboratory of Materials Physics of Ministry of Education, Zhengzhou University, Zhengzhou 450001, China.

[4]School of Power and Mechanical Engineering, Wuhan University, 430072 Wuhan, China.

[5]Max Planck Institute for Polymer Research, Ackermannweg 10, 55128 Mainz, Germany.

[6]Institute of Physics (IA), RWTH Aachen University, Sommerfeldstraße 14, 52074 Aachen, Germany.

*Correspondence: yuehou@whu.edu.cn; houyoumin@whu.edu.cn; yu@physik.rwth-aachen.de; zywang@whu.edu.cn

Inventory of Supplementary Information:

Supplementary Figures 1-22
Supplementary Tables 1 and 2
Supplementary Notes 1 and 2
Supplementary References 1-12

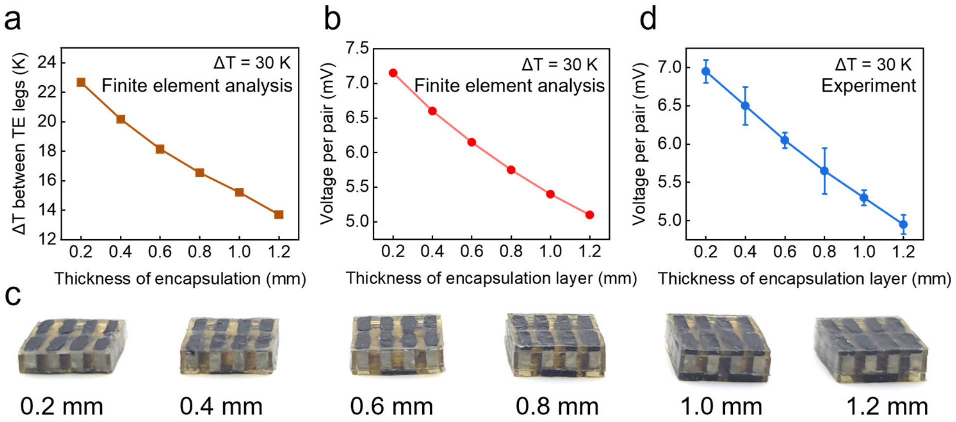

Supplementary Figure 1 a Finite element analysis of ΔT between the upper and lower ends of thermoelectric legs for various encapsulation layer thicknesses. **b** Finite element analysis of open-circuit voltage for thermoelectric devices with different encapsulation layer thicknesses. **c** Optical images of thermoelectric devices with various encapsulation layer thicknesses. **d** The open-circuit voltage of thermoelectric devices with different encapsulation layer thicknesses measured experimentally (the error bar represents the standard deviation, reflecting the degree of data dispersion in three independent experiments).

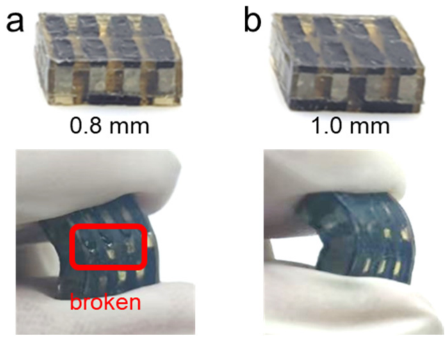

Supplementary Figure 2 a Optical images of thermoelectric devices with 0.8 mm encapsulation layer thicknesses. **b** Optical images of thermoelectric devices with 1 mm encapsulation layer thicknesses.

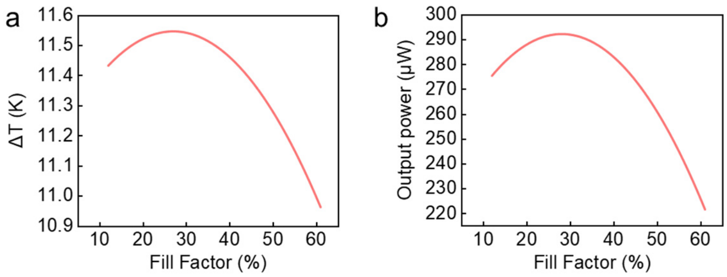

Supplementary Figure 3 a The effect of the filling factor on the actual temperature difference between the upper and lower ends of the thermoelectric leg; **b** The influence of the fill factor on the output power of thermoelectric devices.

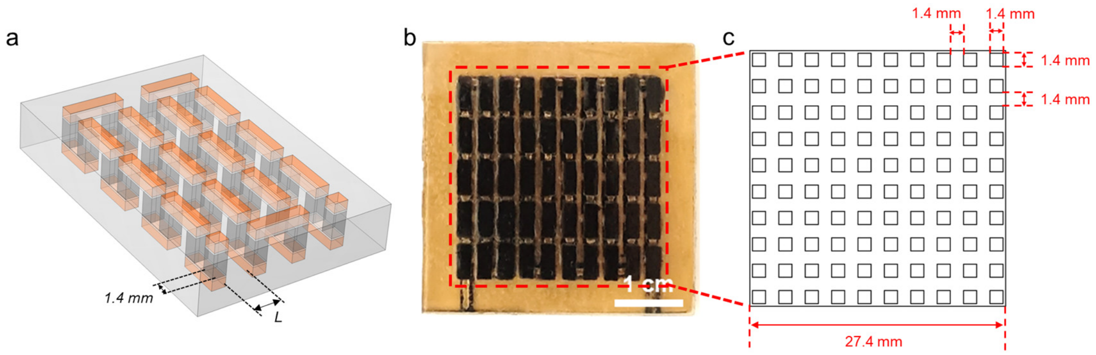

Supplementary Figure 4 a Finite element analysis model of the thermoelectric device for fill factor impact on thermoelectric performance; **b** Optical image of the thermoelectric device with a fill factor of 25%; **c** Top view structural schematic of the thermoelectric device with a fill factor of 25%.

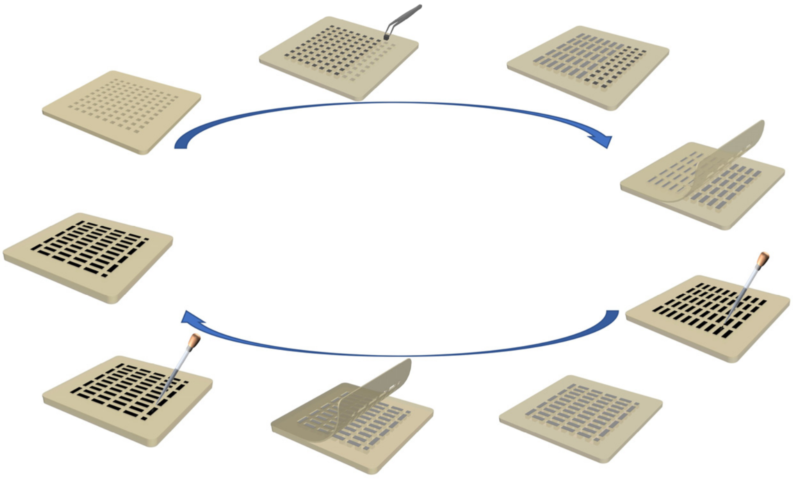

Supplementary Figure 5 Fabrication process for the CD-TED.

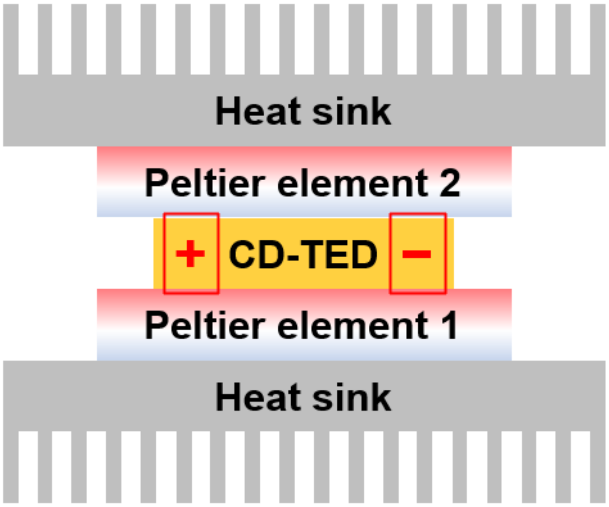

Supplementary Figure 6 Home-made temperature difference application equipment.

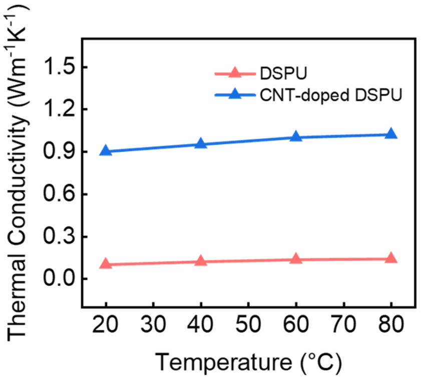

Supplementary Figure 7 The thermal conductivity of DSPU and CNT-doped DSPU

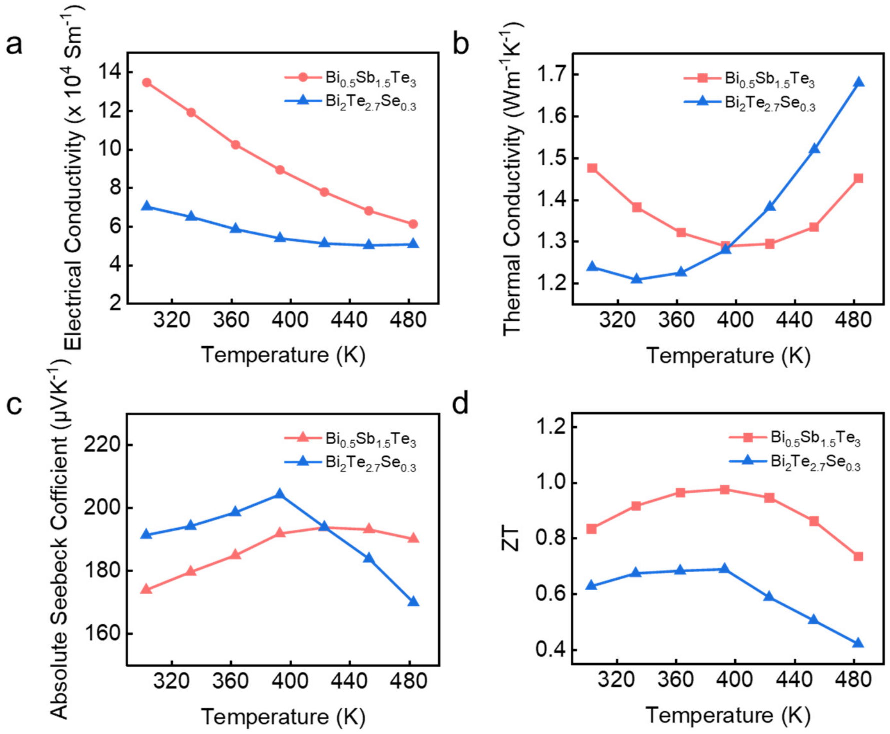

Supplementary Figure 8 Thermoelectric properties of materials. **a** Electrical conductivity; **b** thermal conductivity; **c** absolute Seebeck coefficient; **d** ZT value of $Bi_{0.5}Sb_{1.5}Te_3$ and $Bi_2Te_{2.7}Se_{0.3}$.

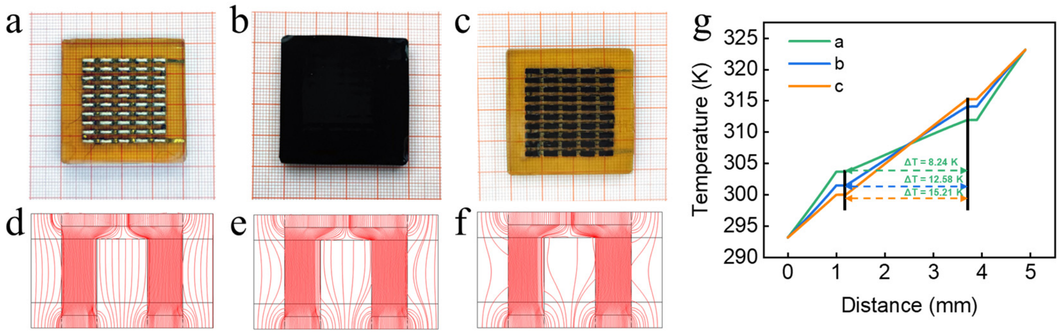

Supplementary Figure 9 **a** Thermoelectric device encapsulated with pure DSPU material; **b** Thermoelectric device homogeneously encapsulated with CNT-doped DSPU material; **c** Thermoelectric device selectively encapsulated with CNT-doped DSPU material; **d-g** Finite element analysis results showing the temperature distribution in the cross-sections of the three thermoelectric devices.

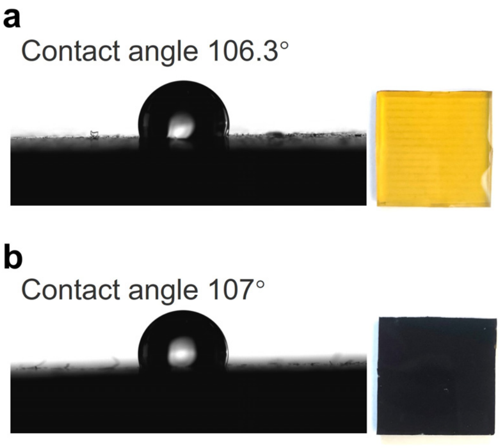

Supplementary Figure 10 Contact angle test of self-healing materials. **a** DSPU; **b** CD.

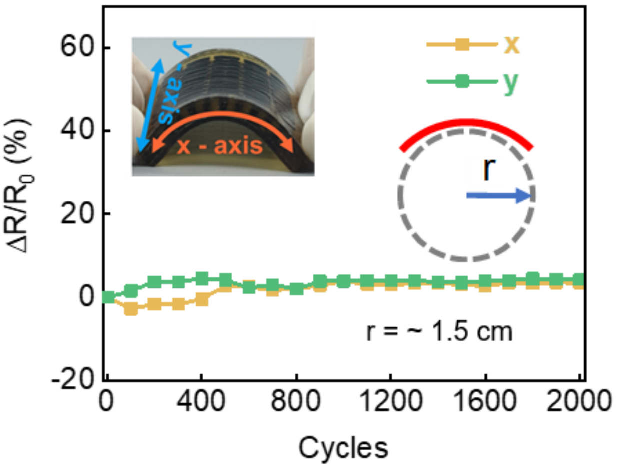

Supplementary Figure 11 Bending cyclic test of the CD-TED showing stable electrical conductivity after bending cycles with bending radius (r) ~1.5 cm.

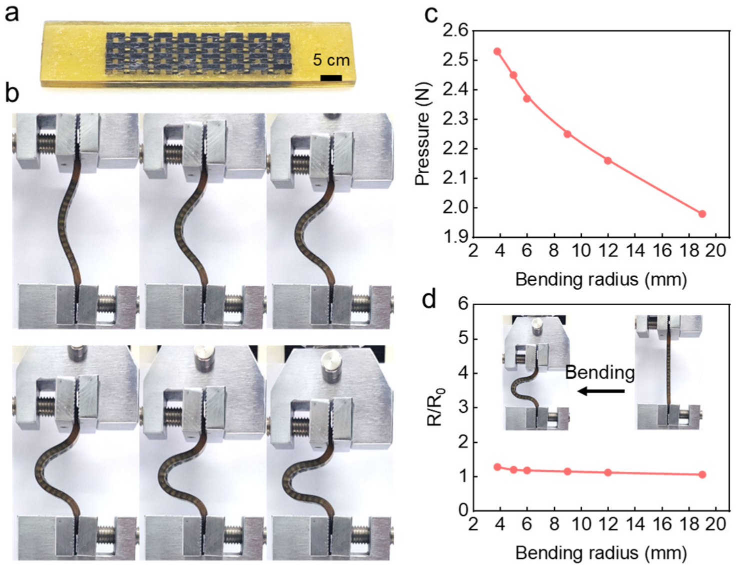

Supplementary Figure 12 Bending tests of the thermoelectric device. **a** The optical image of the thermoelectric device; **b** The optical images of the thermoelectric device at various bending radii; **c** Force required to bend the thermoelectric device at different radii; **d** Relative resistance changes of the thermoelectric device at different bending radii.

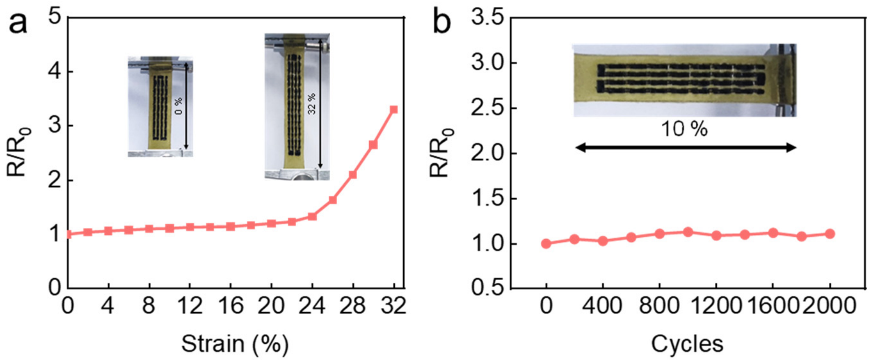

Supplementary Figure 13 Stretchability and reliability of the TED. **a** Variation of electrical resistance with uniaxial strain (0% to 32%) of the thermoelectric device. The inset shows photographs of the flexible thermoelectric device (TED) at 0% and 32% strain. **b** Cyclic tensile testing of the TED, demonstrating the change in electrical conductivity during cyclic stretching at 10% strain.

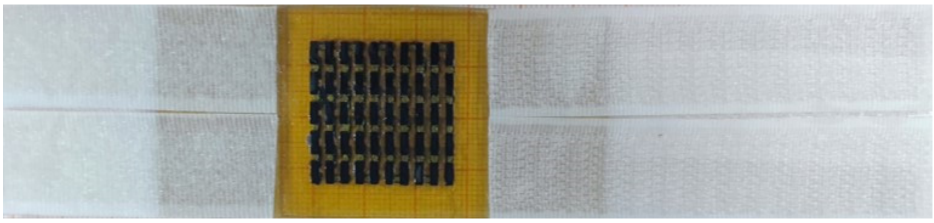

Supplementary Figure 14 The optical image of the wearable CD-TED.

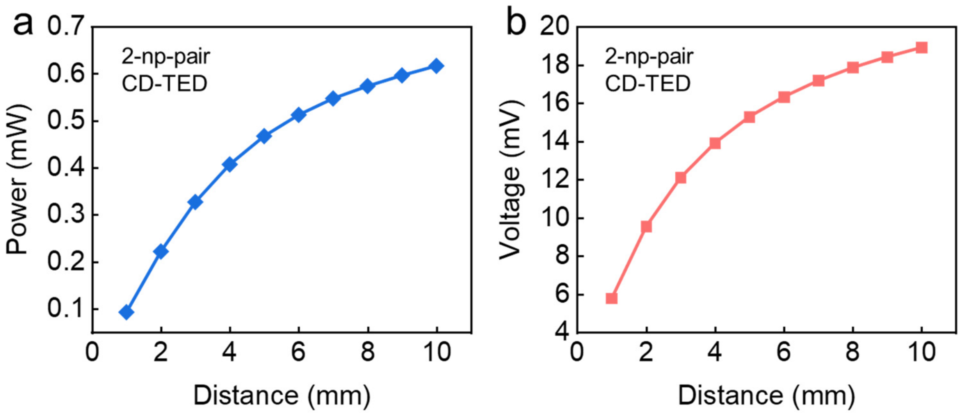

Supplementary Figure 15 The influence of thermoelectric leg height on output power **a** and open circuit voltage **b**.

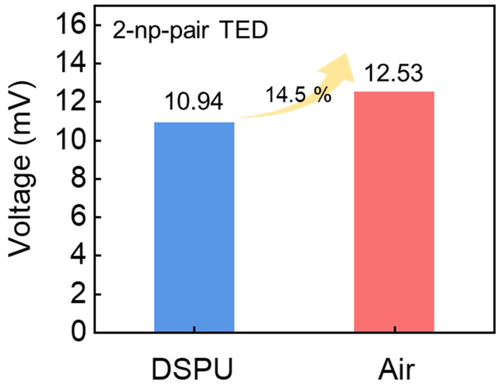

Supplementary Figure 16 The influence of different filling materials on the open circuit voltage of thermoelectric devices: DSPU filling and air filling.

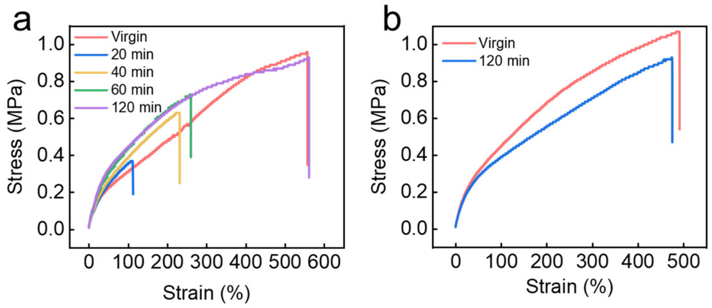

Supplementary Figure 17 **a** Stress-strain curves of DSPU elastomer and **b** DSPU elastomer after two weeks of storage at room temperature (25°C) under different healing times.

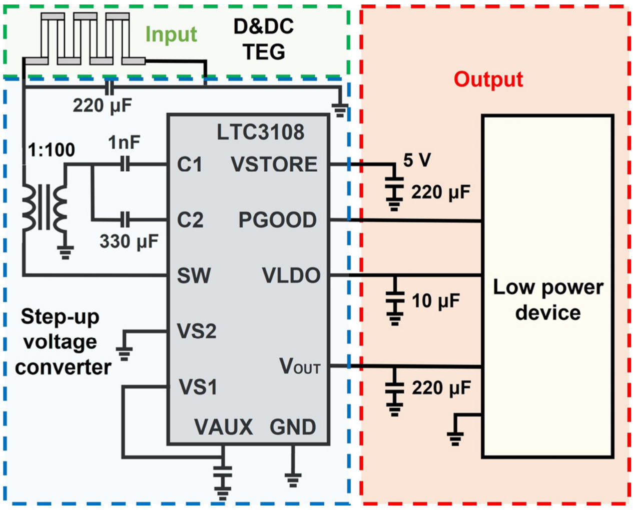

Supplementary Figure 18 Circuit diagram of the PCB for step-up voltage conversion and LED operation.

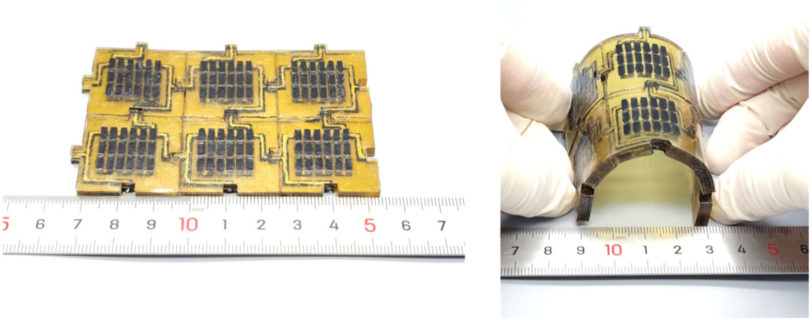

Supplementary Figure 19 The flexibility and adaptability of the newly assembled CD-TED.

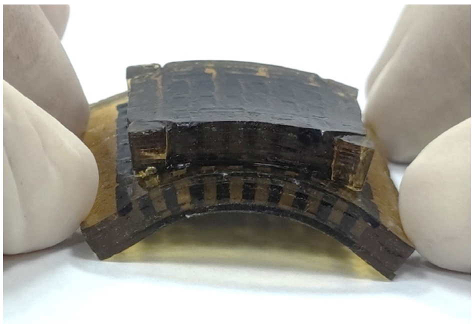

Supplementary Figure 20 Photos of a two-layer tower-like CD-TED when it is bent.

Supplementary Note 1

According to thermoelectric cooling theory, the presence of contact thermal resistance increases the total thermal resistance in the heat flow path, thereby reducing the heat flux density. This leads to a decrease in the heat absorption capacity at the cooling end, which in turn affects the cooling performance of the thermoelectric cooler (TEC). At the same time, an increase in electrode resistance will significantly increase heat loss (Joule heating increase), further reducing the cooling performance.

Specifically, the contact thermal resistance will deteriorate the temperature gradient, and its effective temperature gradient can be expressed as [J. Mater. Chem. A 10, 24985–24994 (2022)]:

$$\nabla T_{eff} = \nabla T \cdot (1 - \frac{R_c^{th}}{R_{total}^{th}})$$

In addition, the contact electrical resistance will also cause a secondary distribution of Joule heat, leading to additional heat generation in the contact area:

$$Q_j = I^2 R_c^{elec}$$

This additional Joule heat can cause local temperature distortion, further affecting the device's performance. At the same time, the degradation of electrical contact will lead to a decrease in the effective Seebeck voltage, which is related to:

$$V_{eff} = V_{Seebeck} - I R_c^{elec}$$

We have performed finite element analysis to investigate the effect of the contact electrical resistance (R_c^{elec}) and the contact thermal resistance (R_c^{th}) on the cooling performance of thermoelectric devices (**Supplementary Figure 21**). The cooling performance of the thermoelectric device gradually decreases with increasing R_c^{elec} or R_c^{th}. These simulation results provide valuable guidance in experimental design and material selection. In future experimental research, we will focus on optimizing contact materials and structures to reduce contact resistance and thermal resistance, thereby improving the cooling efficiency of thermoelectric devices. This will not only enhance the overall performance of the device but also promote the widespread application of thermoelectric devices in practical use.

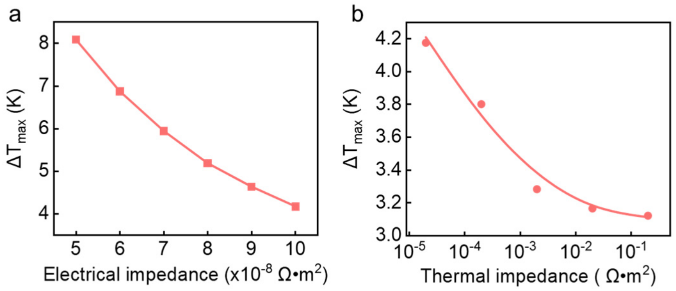

Supplementary Figure 21 **a** The impact of electrical resistance on the maximum cooling capacity of thermoelectric devices; **b** The impact of thermal resistance on the maximum cooling capacity of thermoelectric devices.

Supplementary Note 2

The **Supplementary Figure 22** illustrates the synthesis process of the DSPU elastomer, in which poly(propylene glycol) (PPG) with a molecular weight of 4000 was chosen as the soft segment diol and reacted with isophorone diisocyanate (IPDI) in the presence of the catalyst ditin butyl dilaurate (DBTDL). Among the covalent bonds that can undergo reversible exchange at room temperature, the exchange reaction of aromatic disulfides has significant advantages due to its simplicity and availability. Therefore, 4-aminophenyl disulfide (APDS) containing aromatic disulfide units was selected as the hard segment. In addition, the large number of urea groups formed in the chain can form intermolecular and intramolecular hydrogen bonds at the molecular level, thereby physically cross-linking and forming a supramolecular DSPU polymer network.

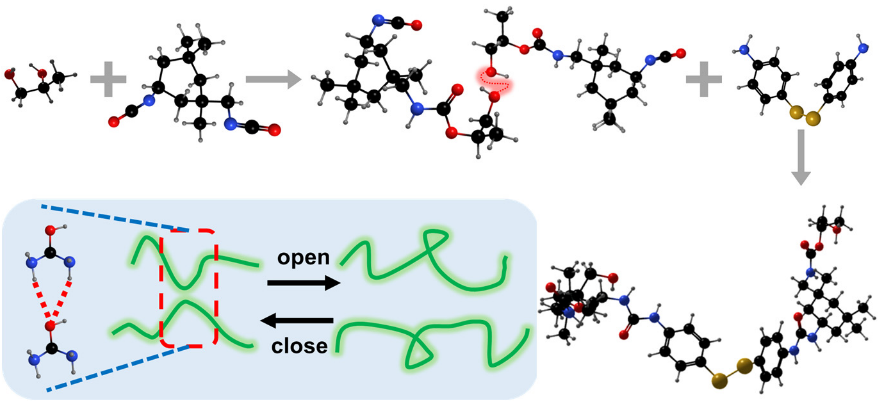

Supplementary Figure 22 Schematic diagram of the synthesis route and self-healing mechanism of self-healing materials.

Supplementary Table 1 Comparison of TE performance with previous flexible TEDs.

Ref.	Strategy	Normalized Seebeck voltage ($\mu V\ K^{-1}\ cm^{-2}$)	Normalized power density ($\mu W\ cm^{-2}\ K^{-2}$)
1	Ag-Ni/PDMS-Bi_2Te_3	12045	0.997
2	Ecoflex-Bi_2Te_3/Sb_2Te_3	6490	0.415
3	EGaIn/PDMS-Bi_2Te_3	2369	0.238
4	Fabric-$Bi_{0.5}Sb_{1.5}Te_3$/$Bi_2Te_{2.7}Se_{0.3}$	139	0.0024
5	Paper-$Bi_{0.5}Sb_{1.5}Te_3$/$Bi_2Te_{2.7}Se_{0.3}$	2257	0.114
6	Liquid alloy/EcoFlex-Sb_2Te_3/Bi_2Te_3	917	0.16
7	Polyimide-Ni doped Bi_2Te_3/$Bi_{0.3}Sb_{1.7}Te_3$	1530	2.17
8	Glass fabric-screen printed Bi_2Te_3 and Sb_2Te_3	600	1.52
9	Polyimine-Bi_2Te_3/Sb_2Te_3	1302	1.08
10	Polyimine/BN-Bi_2Te_3/Sb_2Te_3	1340	1.54
11	CNT	5800	1.864
12	SWCNT	60.34	0.00013
13	This works	4387	3.14

Supplementary Table 2 TE properties of bismuth telluride (Bi_2Te_3) legs.

	Dimension (H×W×D) (mm^3)	Seebeck coefficient (μV/K)	Electrical conductivity (S/m)	Thermal conductivity (W/(m·K))
$Bi_{0.5}Sb_{1.5}Te_3$ (p-type)	2.5×1.4×1.4	174.3	13.55×10^4	1.2-1.4
$Bi_2Sb_{2.7}Te_{0.3}$ (n-type)	2.5×1.4×1.4	-191.5	7.5×10^4	1.2-1.4

Supplementary References

1. Zadan, M., Malakooti, M. H. & Majidi, C. Soft and Stretchable Thermoelectric Generators Enabled by Liquid Metal Elastomer Composites. *ACS Appl. Mater. Interfaces* **12**, 17921–17928 (2020).
2. Yang, Y. *et al.* Stretchable Nanolayered Thermoelectric Energy Harvester on Complex and Dynamic Surfaces. *Nano Lett.* **20**, 4445–4453 (2020).
3. Zhu, P. *et al.* Recyclable, Healable, and Stretchable High-Power Thermoelectric Generator. *Adv. Energy Mater.* **11**, 2100920 (2021).
4. Hou, Y. *et al.* Whole Fabric-Assisted Thermoelectric Devices for Wearable Electronics. *Adv. Sci.* **9**, 2103574 (2022).
5. Kim, S. J., We, J. H. & Cho, B. J. A wearable thermoelectric generator fabricated on a glass fabric. *Energy Environ. Sci.* **7**, 1959 (2014).
6. Zhu, P. *et al.* A self-healable, recyclable, and flexible thermoelectric device for wearable energy harvesting and personal thermal management. *Energy Convers. Manag.* **285**, 117017 (2023).
7. Lee, B. *et al.* High-performance compliant thermoelectric generators with magnetically self-assembled soft heat conductors for self-powered wearable electronics. *Nat. Commun.* **11**, 5948 (2020).
8. Van Toan, N., Kim Tuoi, T. T. & Ono, T. High-performance flexible thermoelectric generator for self-powered wireless BLE sensing systems. *J. Power Sources* **536**, 231504 (2022).
9. Zhang, X. *et al.* Stamp-Like Energy Harvester and Programmable Information Encrypted Display Based on Fully Printable Thermoelectric Devices. *Adv. Mater.* **35**, 2207723 (2023).
10. Jeong, S. H. *et al.* Stretchable Thermoelectric Generators Metallized with Liquid Alloy. *ACS Appl. Mater. Interfaces* **9**, 15791–15797 (2017).
11. Park, K. T. *et al.* Highly Integrated, Wearable Carbon-Nanotube-Yarn-Based Thermoelectric Generators Achieved by Selective Inkjet-Printed Chemical Doping. *Adv. Energy Mater.* **12**, 2200256 (2022).
12. Mytafides, C. K., Tzounis, L., Karalis, G., Formanek, P. & Paipetis, A. S. High-Power All-Carbon Fully Printed and Wearable SWCNT-Based Organic Thermoelectric Generator. *ACS Appl. Mater. Interfaces* **13**, 11151–11165 (2021).
